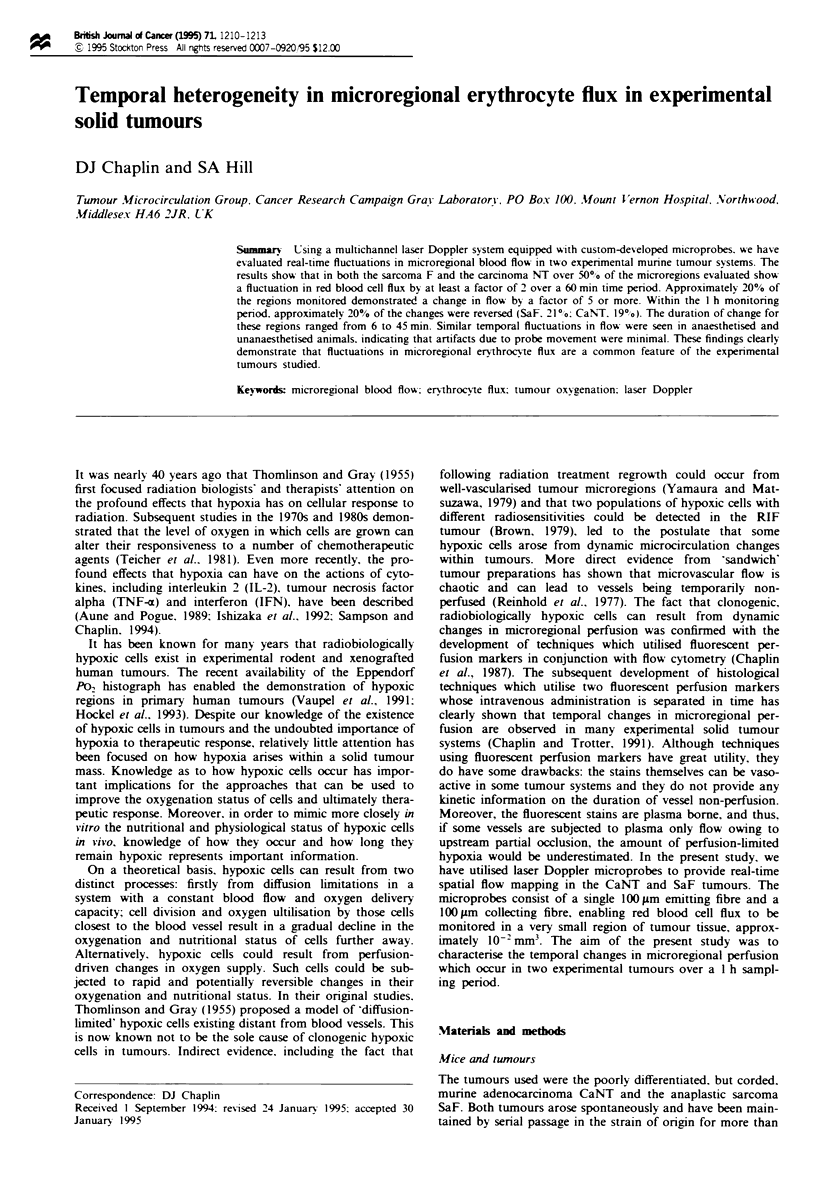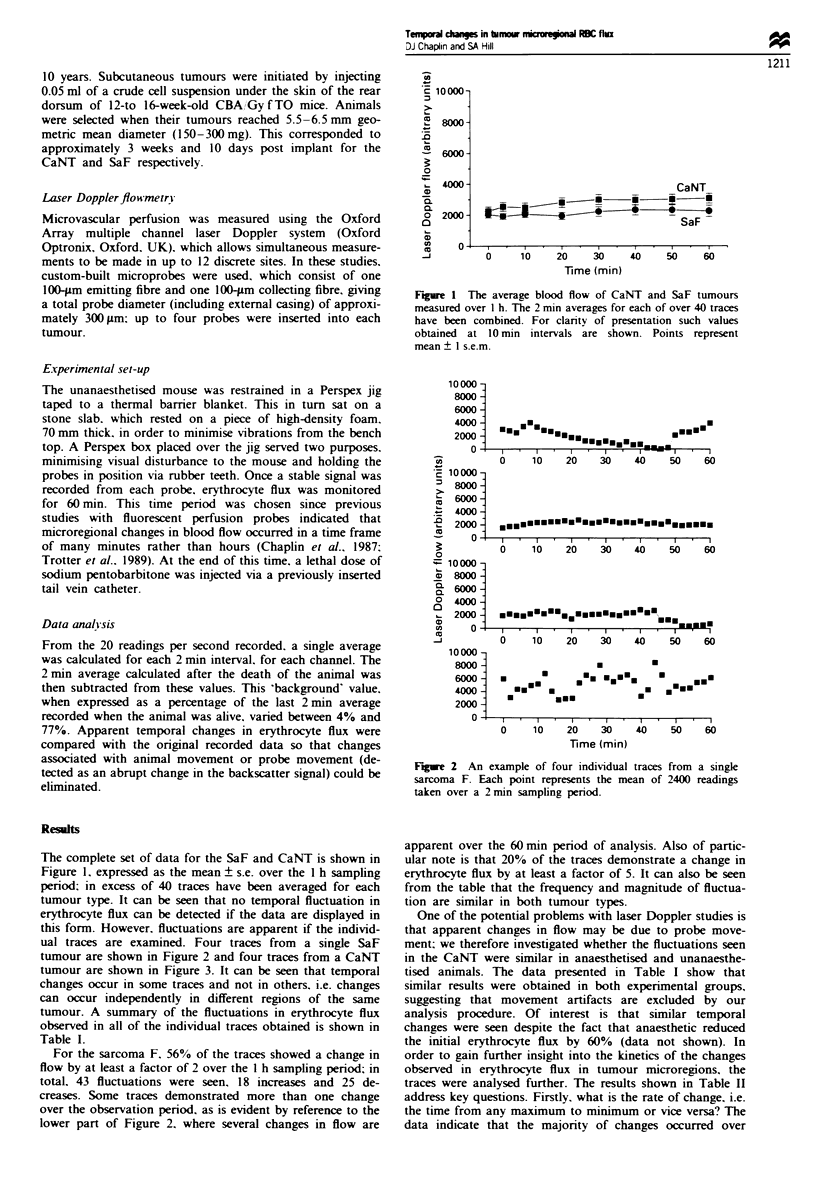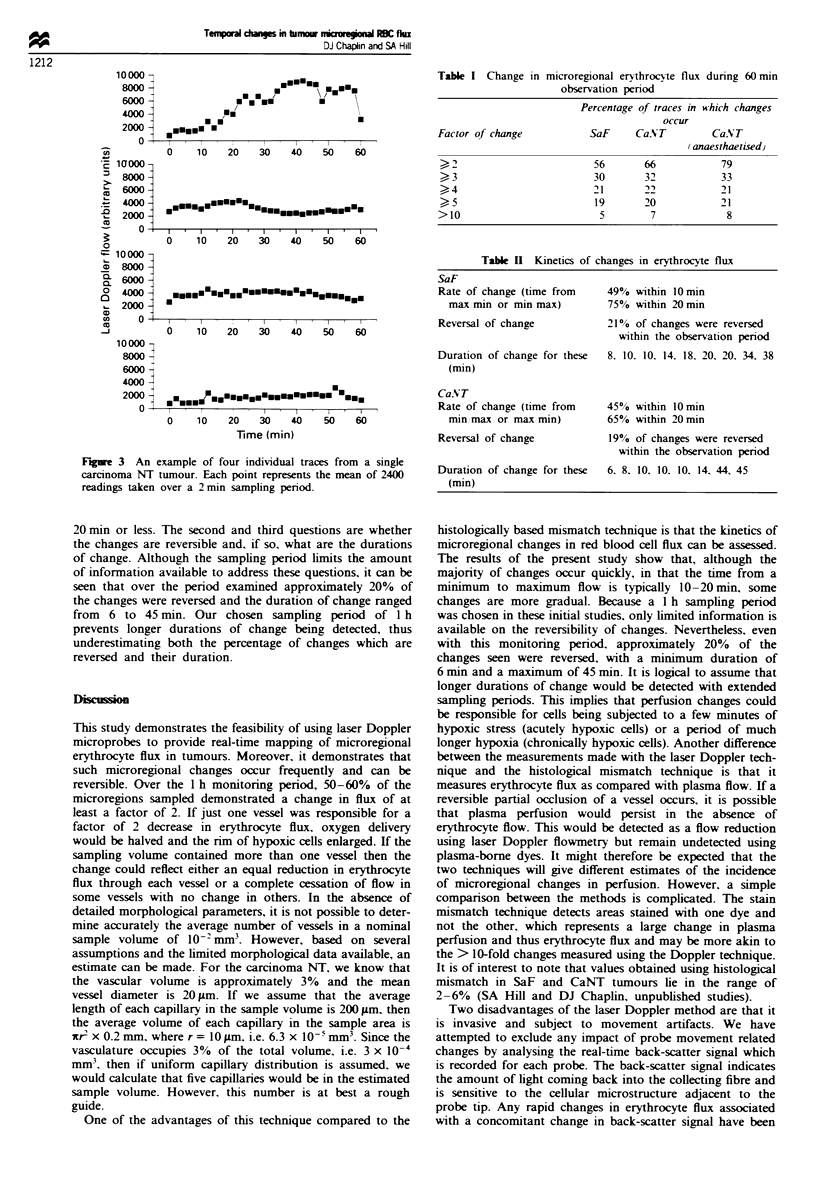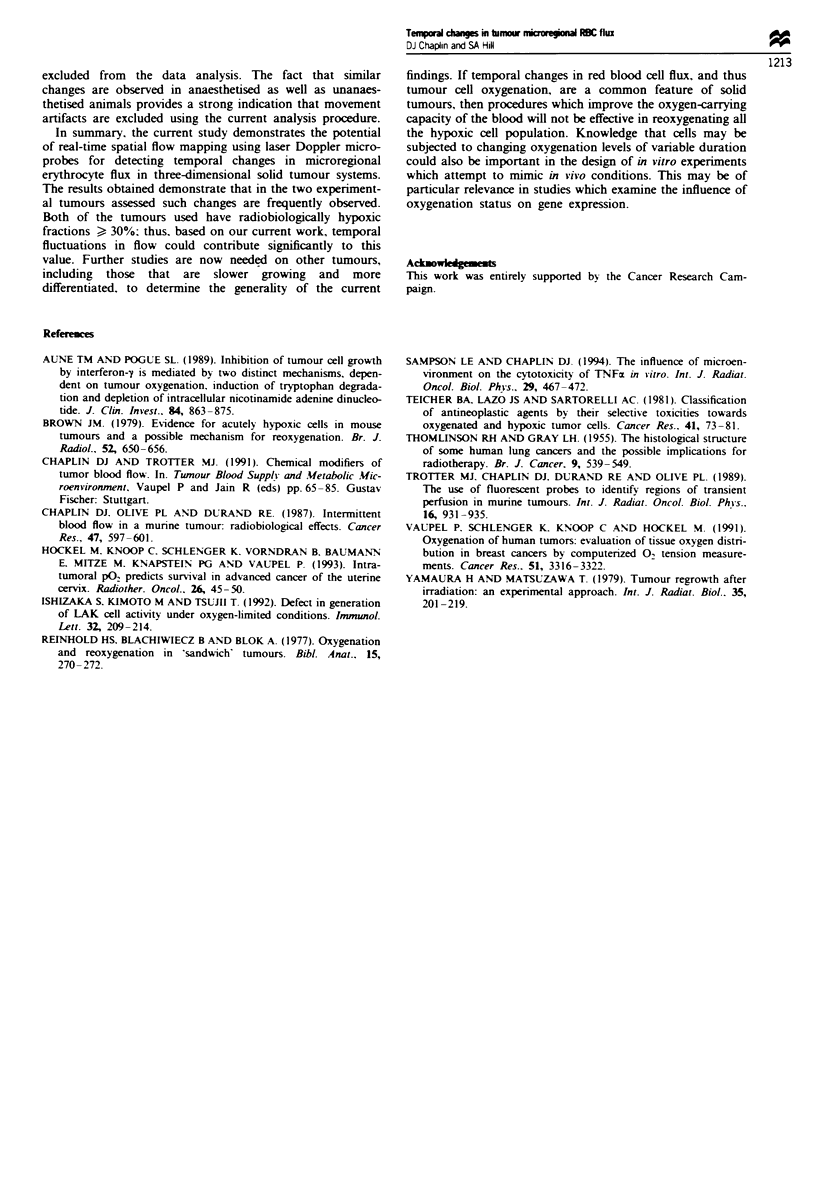# Temporal heterogeneity in microregional erythrocyte flux in experimental solid tumours.

**DOI:** 10.1038/bjc.1995.235

**Published:** 1995-06

**Authors:** D. J. Chaplin, S. A. Hill

**Affiliations:** Tumour Microcirculation Group, Cancer Research Campaign Gray Laboratory, Mount Vernon Hospital, Northwood, Middlesex, UK.

## Abstract

Using a multichannel laser Doppler system equipped with custom-developed microprobes, we have evaluated real-time fluctuations in microregional blood flow in two experimental murine tumour systems. The results show that in both the sarcoma F and the carcinoma NT over 50% of the microregions evaluated show a fluctuation in red blood cell flux by at least a factor of 2 over a 60 min time period. Approximately 20% of the regions monitored demonstrated a change in flow by a factor of 5 or more. Within the 1 h monitoring period, approximately 20% of the changes were reversed (SaF, 21%; CaNT, 19%). The duration of change for these regions ranged from 6 to 45 min. Similar temporal fluctuations in flow were seen in anaesthetised and unanaesthetised animals, indicating that artifacts due to probe movement were minimal. These findings clearly demonstrate that fluctuations in microregional erythrocyte flux are a common feature of the experimental tumours studied.


© 1995 Stockton Press All rights reserved 0007-0920/95 $12.00

### Temporal heterogeneity in microregional erythrocyte flux in experimental solid tumours

DJ Chaplin and SA Hill

*Tumour Microcirculation Group, Cancer Research Campaign Gray Laboratory, PO Box 100, Mount Vernon Hospital, Northwood, Middlesex HA6 2JR, UK*

**Summary** Using a multichannel laser Doppler system equipped with custom-developed microprobes, we have evaluated real-time fluctuations in microregional blood flow in two experimental murine tumour systems. The results show that in both the sarcoma F and the carcinoma NT over 50% of the microregions evaluated show a fluctuation in red blood cell flux by at least a factor of 2 over a 60 min time period. Approximately 20% of the regions monitored demonstrated a change in flow by a factor of 5 or more. Within the 1 h monitoring period, approximately 20% of the changes were reversed (SaF, 21%; CaNT, 19%). The duration of change for these regions ranged from 6 to 45 min. Similar temporal fluctuations in flow were seen in anaesthetised and unanaesthetised animals, indicating that artifacts due to probe movement were minimal. These findings clearly demonstrate that fluctuations in microregional erythrocyte flux are a common feature of the experimental tumours studied.

**Keywords:** microregional blood flow; erythrocyte flux; tumour oxygenation; laser Doppler

It was nearly 40 years ago that Thomlinson and Gray (1955) first focused radiation biologists' and therapists' attention on the profound effects that hypoxia has on cellular response to radiation. Subsequent studies in the 1970s and 1980s demonstrated that the level of oxygen in which cells are grown can alter their responsiveness to a number of chemotherapeutic agents (Teicher *et al.*, 1981). Even more recently, the profound effects that hypoxia can have on the actions of cytokines, including interleukin 2 (IL-2), tumour necrosis factor alpha (TNF-α) and interferon (IFN), have been described (Aune and Pogue, 1989; Ishizaka *et al.*, 1992; Sampson and Chaplin, 1994).

It has been known for many years that radiobiologically hypoxic cells exist in experimental rodent and xenografted human tumours. The recent availability of the Eppendorf $Po_2$ histograph has enabled the demonstration of hypoxic regions in primary human tumours (Vaupel *et al.*, 1991; Hockel *et al.*, 1993). Despite our knowledge of the existence of hypoxic cells in tumours and the undoubted importance of hypoxia to therapeutic response, relatively little attention has been focused on how hypoxia arises within a solid tumour mass. Knowledge as to how hypoxic cells occur has important implications for the approaches that can be used to improve the oxygenation status of cells and ultimately therapeutic response. Moreover, in order to mimic more closely *in vitro* the nutritional and physiological status of hypoxic cells *in vivo*, knowledge of how they occur and how long they remain hypoxic represents important information.

On a theoretical basis, hypoxic cells can result from two distinct processes: firstly from diffusion limitations in a system with a constant blood flow and oxygen delivery capacity; cell division and oxygen ultilisation by those cells closest to the blood vessel result in a gradual decline in the oxygenation and nutritional status of cells further away. Alternatively, hypoxic cells could result from perfusion-driven changes in oxygen supply. Such cells could be subjected to rapid and potentially reversible changes in their oxygenation and nutritional status. In their original studies, Thomlinson and Gray (1955) proposed a model of 'diffusion-limited' hypoxic cells existing distant from blood vessels. This is now known not to be the sole cause of clonogenic hypoxic cells in tumours. Indirect evidence, including the fact that following radiation treatment regrowth could occur from well-vascularised tumour microregions (Yamaura and Matsuzawa, 1979) and that two populations of hypoxic cells with different radiosensitivities could be detected in the RIF tumour (Brown, 1979), led to the postulate that some hypoxic cells arose from dynamic microcirculation changes within tumours. More direct evidence from 'sandwich' tumour preparations has shown that microvascular flow is chaotic and can lead to vessels being temporarily non-perfused (Reinhold *et al.*, 1977). The fact that clonogenic, radiobiologically hypoxic cells can result from dynamic changes in microregional perfusion was confirmed with the development of techniques which utilised fluorescent perfusion markers in conjunction with flow cytometry (Chaplin *et al.*, 1987). The subsequent development of histological techniques which utilise two fluorescent perfusion markers whose intravenous administration is separated in time has clearly shown that temporal changes in microregional perfusion are observed in many experimental solid tumour systems (Chaplin and Trotter, 1991). Although techniques using fluorescent perfusion markers have great utility, they do have some drawbacks: the stains themselves can be vasoactive in some tumour systems and they do not provide any kinetic information on the duration of vessel non-perfusion. Moreover, the fluorescent stains are plasma borne, and thus, if some vessels are subjected to plasma only flow owing to upstream partial occlusion, the amount of perfusion-limited hypoxia would be underestimated. In the present study, we have utilised laser Doppler microprobes to provide real-time spatial flow mapping in the CaNT and SaF tumours. The microprobes consist of a single 100 μm emitting fibre and a 100 μm collecting fibre, enabling red blood cell flux to be monitored in a very small region of tumour tissue, approximately $10^{-2}$ mm$^3$. The aim of the present study was to characterise the temporal changes in microregional perfusion which occur in two experimental tumours over a 1 h sampling period.

### Materials and methods

### *Mice and tumours*

The tumours used were the poorly differentiated, but corded, murine adenocarcinoma CaNT and the anaplastic sarcoma SaF. Both tumours arose spontaneously and have been maintained by serial passage in the strain of origin for more than

Correspondence: DJ Chaplin
Received 1 September 1994; revised 24 January 1995; accepted 30 January 1995

10 years. Subcutaneous tumours were initiated by injecting 0.05 ml of a crude cell suspension under the skin of the rear dorsum of 12-to 16-week-old CBA/Gy f TO mice. Animals were selected when their tumours reached 5.5–6.5 mm geometric mean diameter (150–300 mg). This corresponded to approximately 3 weeks and 10 days post implant for the CaNT and SaF respectively.

*Laser Doppler flowmetry*

Microvascular perfusion was measured using the Oxford Array multiple channel laser Doppler system (Oxford Optronix, Oxford, UK), which allows simultaneous measurements to be made in up to 12 discrete sites. In these studies, custom-built microprobes were used, which consist of one 100-μm emitting fibre and one 100-μm collecting fibre, giving a total probe diameter (including external casing) of approximately 300 μm; up to four probes were inserted into each tumour.

*Experimental set-up*

The unanaesthetised mouse was restrained in a Perspex jig taped to a thermal barrier blanket. This in turn sat on a stone slab, which rested on a piece of high-density foam, 70 mm thick, in order to minimise vibrations from the bench top. A Perspex box placed over the jig served two purposes, minimising visual disturbance to the mouse and holding the probes in position via rubber teeth. Once a stable signal was recorded from each probe, erythrocyte flux was monitored for 60 min. This time period was chosen since previous studies with fluorescent perfusion probes indicated that microregional changes in blood flow occurred in a time frame of many minutes rather than hours (Chaplin *et al.*, 1987; Trotter *et al.*, 1989). At the end of this time, a lethal dose of sodium pentobarbitone was injected via a previously inserted tail vein catheter.

*Data analysis*

From the 20 readings per second recorded, a single average was calculated for each 2 min interval, for each channel. The 2 min average calculated after the death of the animal was then subtracted from these values. This 'background' value, when expressed as a percentage of the last 2 min average recorded when the animal was alive, varied between 4% and 77%. Apparent temporal changes in erythrocyte flux were compared with the original recorded data so that changes associated with animal movement or probe movement (detected as an abrupt change in the backscatter signal) could be eliminated.

**Figure 1** The average blood flow of CaNT and SaF tumours measured over 1 h. The 2 min averages for each of over 40 traces have been combined. For clarity of presentation such values obtained at 10 min intervals are shown. Points represent mean ± 1 s.e.m.

**Figure 2** An example of four individual traces from a single sarcoma F. Each point represents the mean of 2400 readings taken over a 2 min sampling period.

### Results

The complete set of data for the SaF and CaNT is shown in Figure 1, expressed as the mean ± s.e. over the 1 h sampling period; in excess of 40 traces have been averaged for each tumour type. It can be seen that no temporal fluctuation in erythrocyte flux can be detected if the data are displayed in this form. However, fluctuations are apparent if the individual traces are examined. Four traces from a single SaF tumour are shown in Figure 2 and four traces from a CaNT tumour are shown in Figure 3. It can be seen that temporal changes occur in some traces and not in others, i.e. changes can occur independently in different regions of the same tumour. A summary of the fluctuations in erythrocyte flux observed in all of the individual traces obtained is shown in Table I.

For the sarcoma F, 56% of the traces showed a change in flow by at least a factor of 2 over the 1 h sampling period; in total, 43 fluctuations were seen, 18 increases and 25 decreases. Some traces demonstrated more than one change over the observation period, as is evident by reference to the lower part of Figure 2, where several changes in flow are apparent over the 60 min period of analysis. Also of particular note is that 20% of the traces demonstrate a change in erythrocyte flux by at least a factor of 5. It can also be seen from the table that the frequency and magnitude of fluctuation are similar in both tumour types.

One of the potential problems with laser Doppler studies is that apparent changes in flow may be due to probe movement; we therefore investigated whether the fluctuations seen in the CaNT were similar in anaesthetised and unanaesthetised animals. The data presented in Table I show that similar results were obtained in both experimental groups, suggesting that movement artifacts are excluded by our analysis procedure. Of interest is that similar temporal changes were seen despite the fact that anaesthetic reduced the initial erythrocyte flux by 60% (data not shown). In order to gain further insight into the kinetics of the changes observed in erythrocyte flux in tumour microregions, the traces were analysed further. The results shown in Table II address key questions. Firstly, what is the rate of change, i.e. the time from any maximum to minimum or vice versa? The data indicate that the majority of changes occurred over

**Figure 3** An example of four individual traces from a single carcinoma NT tumour. Each point represents the mean of 2400 readings taken over a 2 min sampling period.

**Table I** Change in microregional erythrocyte flux during 60 min observation period

| | Percentage of traces in which changes occur | | |
|---|---|---|---|
| *Factor of change* | *SaF* | *CaNT* | *CaNT (anaesthaetised)* |
| ⩾2 | 56 | 66 | 79 |
| ⩾3 | 30 | 32 | 33 |
| ⩾4 | 21 | 22 | 21 |
| ⩾5 | 19 | 20 | 21 |
| >10 | 5 | 7 | 8 |

**Table II** Kinetics of changes in erythrocyte flux

| | |
|---|---|
| *SaF* | |
| Rate of change (time from max/min or min/max) | 49% within 10 min<br>75% within 20 min |
| Reversal of change | 21% of changes were reversed within the observation period |
| Duration of change for these (min) | 8, 10, 10, 14, 18, 20, 20, 34, 38 |
| *CaNT* | |
| Rate of change (time from min/max or max/min) | 45% within 10 min<br>65% within 20 min |
| Reversal of change | 19% of changes were reversed within the observation period |
| Duration of change for these (min) | 6, 8, 10, 10, 10, 14, 44, 45 |

20 min or less. The second and third questions are whether the changes are reversible and, if so, what are the durations of change. Although the sampling period limits the amount of information available to address these questions, it can be seen that over the period examined approximately 20% of the changes were reversed and the duration of change ranged from 6 to 45 min. Our chosen sampling period of 1 h prevents longer durations of change being detected, thus underestimating both the percentage of changes which are reversed and their duration.

### Discussion

This study demonstrates the feasibility of using laser Doppler microprobes to provide real-time mapping of microregional erythrocyte flux in tumours. Moreover, it demonstrates that such microregional changes occur frequently and can be reversible. Over the 1 h monitoring period, 50–60% of the microregions sampled demonstrated a change in flux of at least a factor of 2. If just one vessel was responsible for a factor of 2 decrease in erythrocyte flux, oxygen delivery would be halved and the rim of hypoxic cells enlarged. If the sampling volume contained more than one vessel then the change could reflect either an equal reduction in erythrocyte flux through each vessel or a complete cessation of flow in some vessels with no change in others. In the absence of detailed morphological parameters, it is not possible to determine accurately the average number of vessels in a nominal sample volume of $10^{-2}$ mm$^3$. However, based on several assumptions and the limited morphological data available, an estimate can be made. For the carcinoma NT, we know that the vascular volume is approximately 3% and the mean vessel diameter is 20 $\mu$m. If we assume that the average length of each capillary in the sample volume is 200 $\mu$m, then the average volume of each capillary in the sample area is $\pi r^2 \times 0.2$ mm, where $r = 10\ \mu$m, i.e. $6.3 \times 10^{-5}$ mm$^3$. Since the vasculature occupies 3% of the total volume, i.e. $3 \times 10^{-4}$ mm$^3$, then if uniform capillary distribution is assumed, we would calculate that five capillaries would be in the estimated sample volume. However, this number is at best a rough guide.

One of the advantages of this technique compared to the histologically based mismatch technique is that the kinetics of microregional changes in red blood cell flux can be assessed. The results of the present study show that, although the majority of changes occur quickly, in that the time from a minimum to maximum flow is typically 10–20 min, some changes are more gradual. Because a 1 h sampling period was chosen in these initial studies, only limited information is available on the reversibility of changes. Nevertheless, even with this monitoring period, approximately 20% of the changes seen were reversed, with a minimum duration of 6 min and a maximum of 45 min. It is logical to assume that longer durations of change would be detected with extended sampling periods. This implies that perfusion changes could be responsible for cells being subjected to a few minutes of hypoxic stress (acutely hypoxic cells) or a period of much longer hypoxia (chronically hypoxic cells). Another difference between the measurements made with the laser Doppler technique and the histological mismatch technique is that it measures erythrocyte flux as compared with plasma flow. If a reversible partial occlusion of a vessel occurs, it is possible that plasma perfusion would persist in the absence of erythrocyte flow. This would be detected as a flow reduction using laser Doppler flowmetry but remain undetected using plasma-borne dyes. It might therefore be expected that the two techniques will give different estimates of the incidence of microregional changes in perfusion. However, a simple comparison between the methods is complicated. The stain mismatch technique detects areas stained with one dye and not the other, which represents a large change in plasma perfusion and thus erythrocyte flux and may be more akin to the >10-fold changes measured using the Doppler technique. It is of interest to note that values obtained using histological mismatch in SaF and CaNT tumours lie in the range of 2–6% (SA Hill and DJ Chaplin, unpublished studies).

Two disadvantages of the laser Doppler method are that it is invasive and subject to movement artifacts. We have attempted to exclude any impact of probe movement related changes by analysing the real-time back-scatter signal which is recorded for each probe. The back-scatter signal indicates the amount of light coming back into the collecting fibre and is sensitive to the cellular microstructure adjacent to the probe tip. Any rapid changes in erythrocyte flux associated with a concomitant change in back-scatter signal have been excluded from the data analysis. The fact that similar changes are observed in anaesthetised as well as unanaesthetised animals provides a strong indication that movement artifacts are excluded using the current analysis procedure.

In summary, the current study demonstrates the potential of real-time spatial flow mapping using laser Doppler microprobes for detecting temporal changes in microregional erythrocyte flux in three-dimensional solid tumour systems. The results obtained demonstrate that in the two experimental tumours assessed such changes are frequently observed. Both of the tumours used have radiobiologically hypoxic fractions $\geqslant 30\%$; thus, based on our current work, temporal fluctuations in flow could contribute significantly to this value. Further studies are now needed on other tumours, including those that are slower growing and more differentiated, to determine the generality of the current findings. If temporal changes in red blood cell flux, and thus tumour cell oxygenation, are a common feature of solid tumours, then procedures which improve the oxygen-carrying capacity of the blood will not be effective in reoxygenating all the hypoxic cell population. Knowledge that cells may be subjected to changing oxygenation levels of variable duration could also be important in the design of *in vitro* experiments which attempt to mimic *in vivo* conditions. This may be of particular relevance in studies which examine the influence of oxygenation status on gene expression.

**Acknowledgements**

This work was entirely supported by the Cancer Research Campaign.

**References**

AUNE TM AND POGUE SL. (1989). Inhibition of tumour cell growth by interferon-γ is mediated by two distinct mechanisms, dependent on tumour oxygenation, induction of tryptophan degradation and depletion of intracellular nicotinamide adenine dinucleotide. *J. Clin. Invest.*, **84**, 863-875.

BROWN JM. (1979). Evidence for acutely hypoxic cells in mouse tumours and a possible mechanism for reoxygenation. *Br. J. Radiol.*, **52**, 650-656.

CHAPLIN DJ AND TROTTER MJ. (1991). Chemical modifiers of tumor blood flow. In. *Tumour Blood Supply and Metabolic Microenvironment*, Vaupel P and Jain R (eds) pp. 65-85. Gustav Fischer: Stuttgart.

CHAPLIN DJ, OLIVE PL AND DURAND RE. (1987). Intermittent blood flow in a murine tumour: radiobiological effects. *Cancer Res.*, **47**, 597-601.

HOCKEL M, KNOOP C, SCHLENGER K, VORNDRAN B, BAUMANN E, MITZE M, KNAPSTEIN PG AND VAUPEL P. (1993). Intratumoral $pO_2$ predicts survival in advanced cancer of the uterine cervix. *Radiother. Oncol.*, **26**, 45-50.

ISHIZAKA S, KIMOTO M AND TSUJII T. (1992). Defect in generation of LAK cell activity under oxygen-limited conditions. *Immunol. Lett.* **32**, 209-214.

REINHOLD HS, BLACHIWIECZ B AND BLOK A. (1977). Oxygenation and reoxygenation in 'sandwich' tumours. *Bibl. Anat.*, **15**, 270-272.

SAMPSON LE AND CHAPLIN DJ. (1994). The influence of microenvironment on the cytotoxicity of TNFα *in vitro*. *Int. J. Radiat. Oncol. Biol. Phys.*, **29**, 467-472.

TEICHER BA, LAZO JS AND SARTORELLI AC. (1981). Classification of antineoplastic agents by their selective toxicities towards oxygenated and hypoxic tumor cells. *Cancer Res.*, **41**, 73-81.

THOMLINSON RH AND GRAY LH. (1955). The histological structure of some human lung cancers and the possible implications for radiotherapy. *Br. J. Cancer*, **9**, 539-549.

TROTTER MJ, CHAPLIN DJ, DURAND RE AND OLIVE PL. (1989). The use of fluorescent probes to identify regions of transient perfusion in murine tumours. *Int. J. Radiat. Oncol. Biol. Phys.*, **16**, 931-935.

VAUPEL P, SCHLENGER K, KNOOP C AND HOCKEL M. (1991). Oxygenation of human tumors: evaluation of tissue oxygen distribution in breast cancers by computerized $O_2$ tension measurements. *Cancer Res.*, **51**, 3316-3322.

YAMAURA H AND MATSUZAWA T. (1979). Tumour regrowth after irradiation: an experimental approach. *Int. J. Radiat. Biol.*, **35**, 201-219.